# Incorporation of Ti^4+^ into the channel of hydroxyapatite by loading of Ti and post-calcination

**DOI:** 10.1039/d6ra08560a

**Published:** 2026-10-26

**Authors:** Xiao Chen, Kanji Saito, Masataka Ogasawara, Sumio Kato

**Affiliations:** a Department of Materials Science, Graduate School of Engineering Science, Akita University 1-1 Tegatagakuen-machi Akita-shi Akita 010-8502 Japan saitok@gipc.akita-u.ac.jp katos@gipc.akita-u.ac.jp; b Kagami Memorial Research Institute for Materials Science and Technology, Waseda University 2-8-26 Nishiwaseda Shinjuku-ku Tokyo 169-0054 Japan

## Abstract

Synthesis of hydroxyapatite (Ca_10_(PO_4_)_6_(OH)_2_, HAp) containing titanium (Ti) in the tetravalent state in its channel was successfully demonstrated by employing a post-treatment involving impregnation with a metal alkoxide followed by heat treatment, together with Rietveld refinement. Infrared spectroscopic analyses suggested that the excess positive charge arising from the cation incorporation was compensated for by proton dissociation from hydroxide ions located in the channel, resulting in the formation of titanium oxo groups in the channel. The incorporation of Ti ions into the interstitial site located in the channel resulted in light absorption characteristics distinct from those typically observed for isomorphous substitution, suggesting broadening of the potential properties of HAp-based materials owing to the expansion of their compositional and structural diversity.

## Abstract 2

Ti^4+^ was successfully incorporated into hydroxyapatite channel *via* impregnation and heating, representing the first example of hydroxyapatite containing channel metal ions with a valence of +4 or higher.

## Graphical abstract

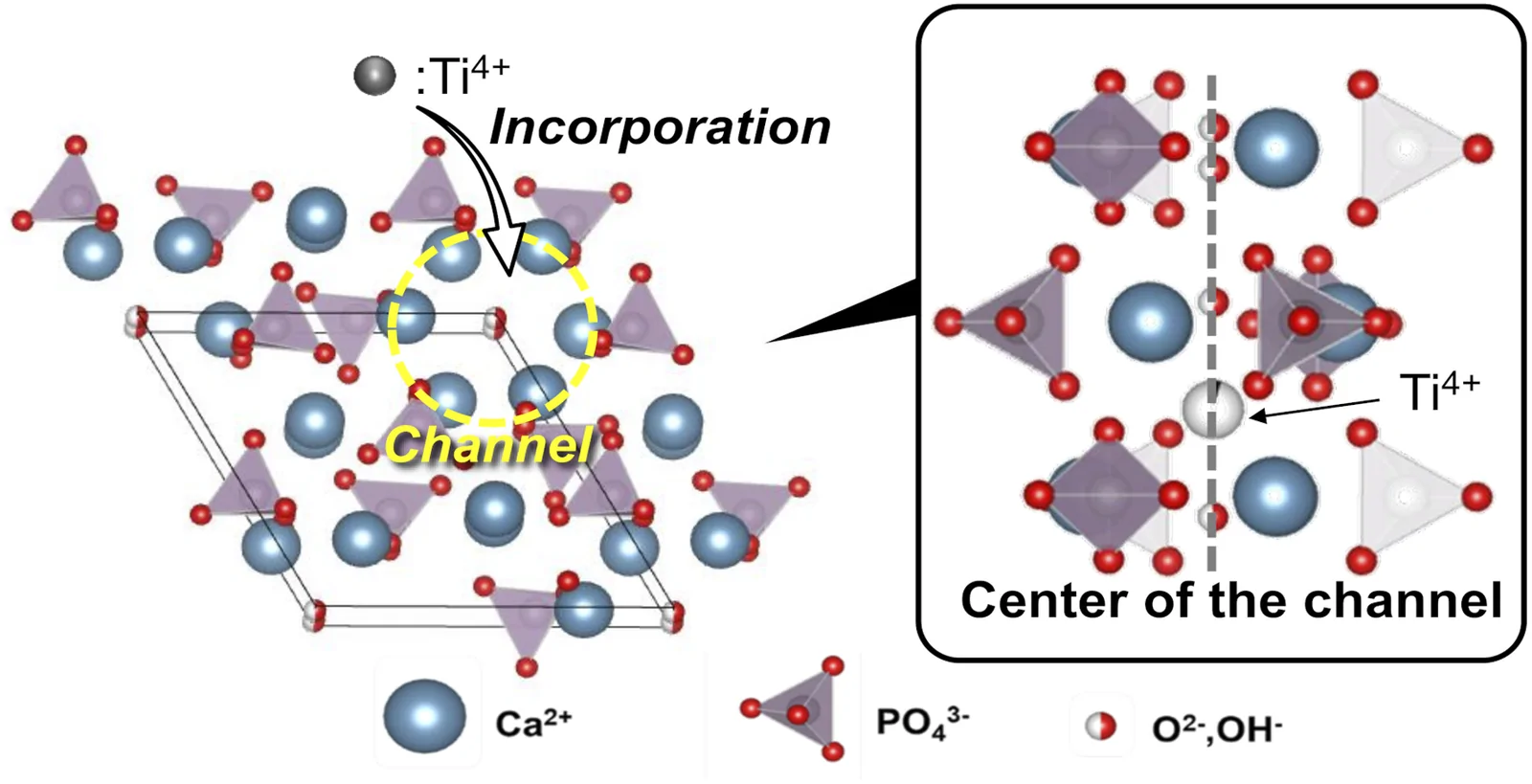

## 1 Introduction

Besides its importance as the principal inorganic constituent of bones and teeth, hydroxyapatite (HAp) has attracted considerable attention as a functional inorganic material for applications such as phosphors,^1–3^ ion exchangers^4–6^ and catalysts/catalyst supports^7–10^ owing to its structural stability, biocompatibility, multiple cation sites enabling diverse cation substitution, acid-based bifunctionality and strong interactions with metals. Isomorphous substitution of HAp has been examined not only from the fundamental perspective of its structure, composition and synthesis, but also from the practical viewpoint of imparting or modifying material functionalities. Substitution of Ca by Na is commonly observed in both natural and synthetic situations.^11,12^ On the other hand, examples of intentionally introduced guest metal ions include alkaline earth metals (*e.g.*, Sr and Ba)^13,14^ and rare earth metals (*e.g.*, Eu and Tb)^15–18^ for tuned affinity with gas molecules and for controlling luminescent properties, respectively. In addition to these large cations, other cations of such transition metals as Cu and Fe have also been selected as substituents in designing dielectric, magnetic and optical characteristics.^19–21^ Cations with higher valences (*e.g.* Ti^4+^ (ref. 22–24) and V^5+^ (ref. 25)) have also attracted attention as guest metals. In addition to calcium substitution, phosphorus and anion substitutions have also been investigated.^26^ HAp in which phosphate was substituted by selenite^27^ is one such example. Whether the desired site is occupied by guest cations is often a matter for debate, however.

In the HAp structure, the channel surrounded by Ca ions provides a site that can accommodate metal ions together with hydroxide ions. HAp containing metal cations in the channel has been synthesized,^28–32^ and has led in some cases to material characteristics that differ from those achieved through conventional isomorphous substitution. HAp containing copper cations in the channel exhibited a vivid red color,^33^ providing a good example of unique characteristics in the case of interstitial site modification. To the best of our knowledge, however, no examples of the synthesis and crystal structural refinement of HAp containing guest cations with oxidation states of +4 or higher in the channel have been reported, although the incorporation of Ti ions into the channel has previously been inferred from changes in the lattice parameters.^34^ The limited number of guest metal ions incorporated by conventional synthetic methods makes it difficult to identify their incorporation sites.

In addition to the direct synthesis of HAp containing cations in the channel, moreover, incorporation of guest metals into the channel of pre-synthesized HAp has been developed by our group.^35–41^ It has been suggested that the excess positive charge arising from the cation incorporation is compensated for by proton dissociation from the hydroxide ions in the channel,^41^ resulting in the formation of metal–oxygen moieties in the channel.^33,36,42,43^ We demonstrated that Zn with a higher content was successfully incorporated into the channel of HAp in the form of [O–Zn–O] units benefiting from the post-synthetic methodology when compared to conventional precipitation methods. In addition, it was revealed that the incorporated Zn ions were deposited as ZnO particles onto the host HAp particles,^40^ a finding inspired by a previous study on reversible incorporation/deposition behavior of guest cations into the channel depending on water content in the atmosphere.^35,41^ Taking advantage of the homogeneous distribution (large surface area) of the deposited ZnO, the resulting composite exhibited higher photocatalytic activity for dye decomposition when compared to that prepared by conventional impregnation methods, suggesting the usefulness of this methodology in designing HAp-based composite materials applicable to various fields including catalysis. In this study, we investigated the synthesis and crystal structural refinement of HAp containing Ti^4+^ as the highest-valent guest cation in the channel reported so far, as well as the deposition of Ti as an oxide from the channel.

## 2 Experimental

### 2.1 Materials

Ca(CH_3_COO)_2_·H_2_O (99.0%), KH_2_PO_4_ (99.0%) and KBr were purchased from Wako Pure Chemical Industries, Ltd. HNO_3_ (60.0%), KOH (85.0%) and titanium(iv) tetraisopropoxide (Ti[(CH_3_)_2_CHO]_4_, TTIP, ≥95.0%) were supplied by Nacalai Tesque, Ltd.

### 2.2 Materials and methods

#### 2.2.1 Synthesis of hydroxyapatite containing Ti in its channel

HAp containing Ti in the channel was synthesized by applying a post-treatment method we have developed. HAp was synthesized by a precipitation method according to a previously reported procedure as follows. Ca(CH_3_COO)_2_·H_2_O and KH_2_PO_4_ were dissolved in distilled water at a molar ratio of 9.8 : 6 using 4 mol L^−1^ HNO_3_. The pH was adjusted to 12.5 with 2 mol L^−1^ KOH to form a precipitate, which was aged at 60 °C for 24 h, washed with water, and dried at 60 °C without any subsequent heat treatment. The resulting HAp powder was impregnated with an ethanolic solution of TTIP in a molar ratio of Ca : Ti = 10 : *x* (*x* = 0–0.2) and dried at 60 °C. The powder was pressed into a pellet, heated for 3 h at 850 or 900 °C in N_2_ at a dewpoint below −50 °C and cooled to obtain Ca_10_(PO_4_)_6_Ti_*x*_O_*y*_H_*z*_ (Ti_*x*_-HAp). To study the deposition behaviors of Ti ions in the channel, the Ti_0.15_-HAp, which was obtained by heat treatment at 850 °C, was annealed at *T* °C (*T* = 800–1000) in air for 12 h.

#### 2.2.2 Synthesis of Ti-substituted hydroxyapatite

Ti-substituted hydroxyapatite was synthesized according to the method of the previous report.^22^ Ca(NO_3_)_2_·4H_2_O was dissolved in decarbonated water, followed by the addition of an aqueous Ti(SO_4_)_2_ solution to achieve a molar ratio of (Ca + Ti) : Ti = 10 : *y* (*y* = 0–0.15). H_3_PO_4_ solution was then added dropwise under stirring. The pH was adjusted to 9 using an aqueous ammonia solution, and the suspension was purged with argon before sealing. The precipitate was aged at 100 °C for 6 h, washed with distilled water, filtered, and dried at 60 °C to obtain Ca_10−*y*_Ti_*y*_-HAp.

### 2.3 Characterization

Phase determination of the products was performed by X-ray diffraction (XRD) using a Rigaku Ultima IV diffractometer with Ni-filtered Cu-Kα radiation (*λ* = 0.15418 nm) operated at 40 kV and 40 mA. The diffraction patterns were collected over a 2*θ* range of 10°–60° or 10°–130° at a scanning rate of 10° min^−1^ or 2° min^−1^, respectively. The XRD patterns were analyzed using PDXL2 software, and the lattice parameters were refined by whole-powder pattern fitting (WPPF) using the Pawley method. A B-spline function was used for background fitting, and a split pseudo-Voigt function was used for peak-profile fitting. Each sample was subjected to XRD measurement once, and the corresponding lattice parameters and unit cell volume were calculated from the obtained diffraction data as described above. Infrared (IR) spectra were recorded by the KBr disk method using a JASCO FT/IR-4700 spectrophotometer. Raman spectra were recorded using a Renishaw inVia reflex spectrometer with a 532 nm laser. Ultraviolet-visible diffuse reflectance (UV-vis DR) spectra were obtained using a JASCO V-750 spectrophotometer equipped with an integrating sphere ISV-922. X-ray photoelectron spectroscopy (XPS) measurements were performed using a KRATOS AXIS-ULTRA spectrometer with monochromated Al Kα radiation. SEM–EDS measurements were performed using a Hitachi High-Technologies SU-70 microscope at an accelerating voltage of 15.0 kV. The samples were mounted on the specimen holder using carbon tape.

## 3 Results and discussion

Fig. 1 shows the XRD patterns of Ti_*x*_-HAp obtained by calcination of HAp impregnated with varying amounts of TTIP at 850 °C. For *x* = 0–0.15, all the diffraction lines were assigned to the apatite phase. For *x* = 0.2, on the other hand, in addition to the diffraction lines ascribable to the apatite phase, diffraction lines attributable to Ca_3_(PO_4_)_2_ and a weak line at 2*θ* = 47.6° assignable to orthorhombic CaTiO_3_ (JCPDS #42-0423), the stable polymorph at the employed temperature,^44^ were observed, indicating a collapse of the host apatite structure.^45^ These results suggest a maximum *x* of 0.15 in the case of 850 °C. Despite the positive effect of an increase in the calcination temperature on the amount of a guest metal cation to be diffused into the channel,^46^ calcination at 900 °C resulted in the formation of the above-mentioned by-products at *x* = 0.15 (Fig. S1), while no by-product formation was observed for a calcination temperature of 850 °C, indicating a negative effect of the loaded Ti species on the thermal stability of HAp. Therefore, we focus hereafter on the samples heat-treated at 850 °C.

**Fig. 1:**
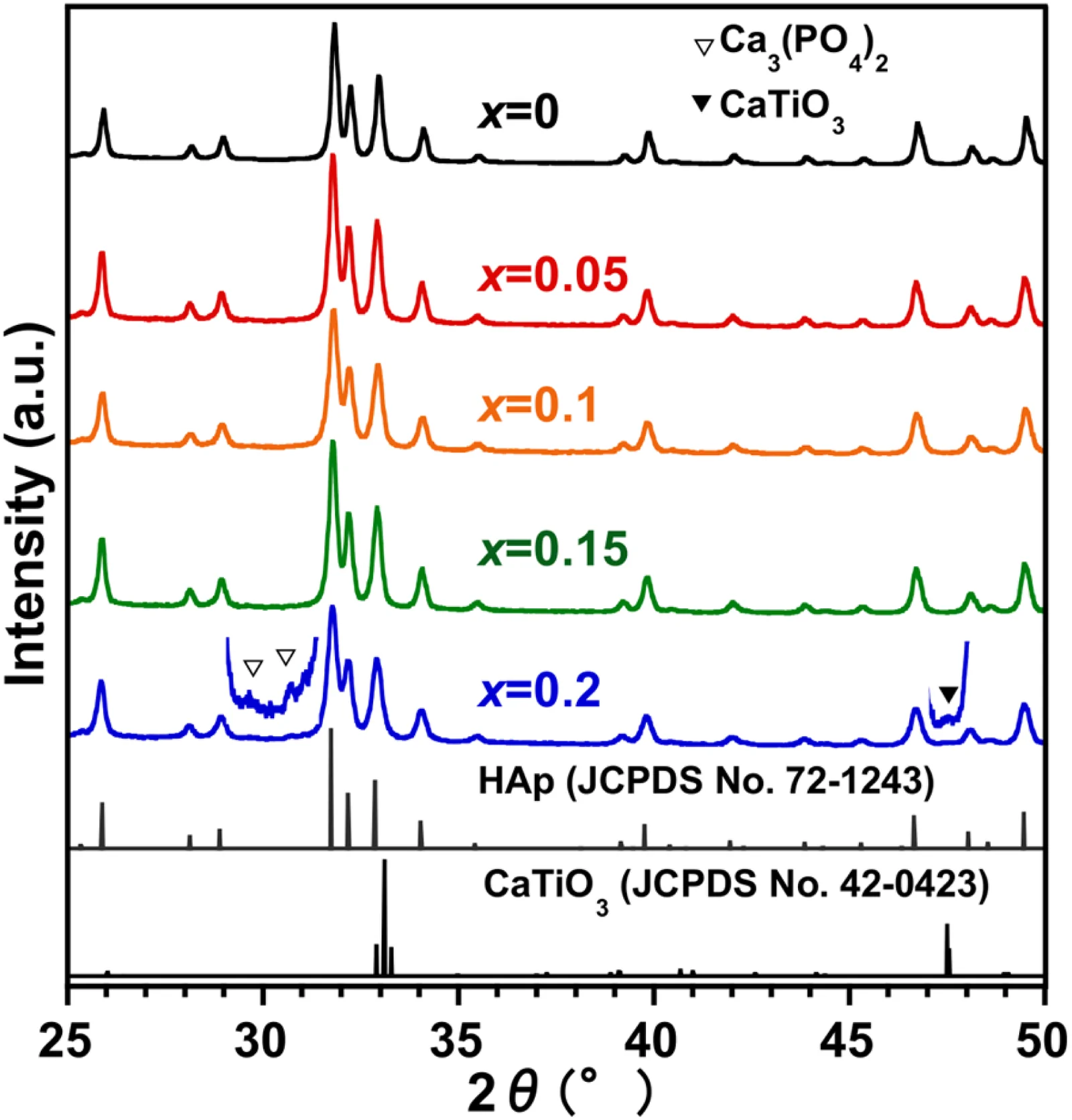
XRD patterns of Ti_*x*_-HAp obtained after heat treatment at 850 °C.

Fig. 2 shows the lattice parameters and unit cell volume of Ti_*x*_-HAp (Table S1) as a function of Ti content (*x*). While the lattice parameter *a* remained approximately constant, *c* increased with increases in the Ti content (*x*) (Fig. 2a), leading to an increase in the unit cell volume (Fig. 2b). Given that the unit cell volume increased when transition metal cations were incorporated into the channel,^35,40^ the expansion of the unit cell volume with *x* suggests incorporation of Ti ions into the channel. It should be noted here that both the lattice parameters (*a* and *c*) and the unit cell volume of HAp decreased with Ti content in the case of isomorphous substitution^47^ because of the smaller ionic radius of Ti^4+^ (0.605 Å) compared with that of Ca^2+^ (0.100 Å).^48^ This relationship between the lattice parameters/unit cell volume and Ti content was also observed for Ca_10−*y*_Ti_*y*_-HAp (Table S1), which were synthesized according to the method of the previous report on Ti-substituted HAp^22^ and were demonstrated to be single phase hydroxyapatites based on the XRD patterns (Fig. S2), confirming the successful formation of reference Ti-substituted HAp samples for comparison with the results of channel modification. It should be noted that, for the products with the highest nominal Ti content that were obtained as single phases, the average Ti/Ca molar ratios estimated by SEM–EDS (Fig. S3 and S4) were somewhat higher (0.021 and 0.018 for Ti_0.15_-HAp and Ca_9.85_Ti_0.15_-HAp, respectively) than the nominal value (0.015) for both products (Table S3). The discrepancy between the nominal and experimental values for Ti_0.15_-HAp is likely associated with intraparticle segregation of Ti, as discussed below.

**Fig. 2:**
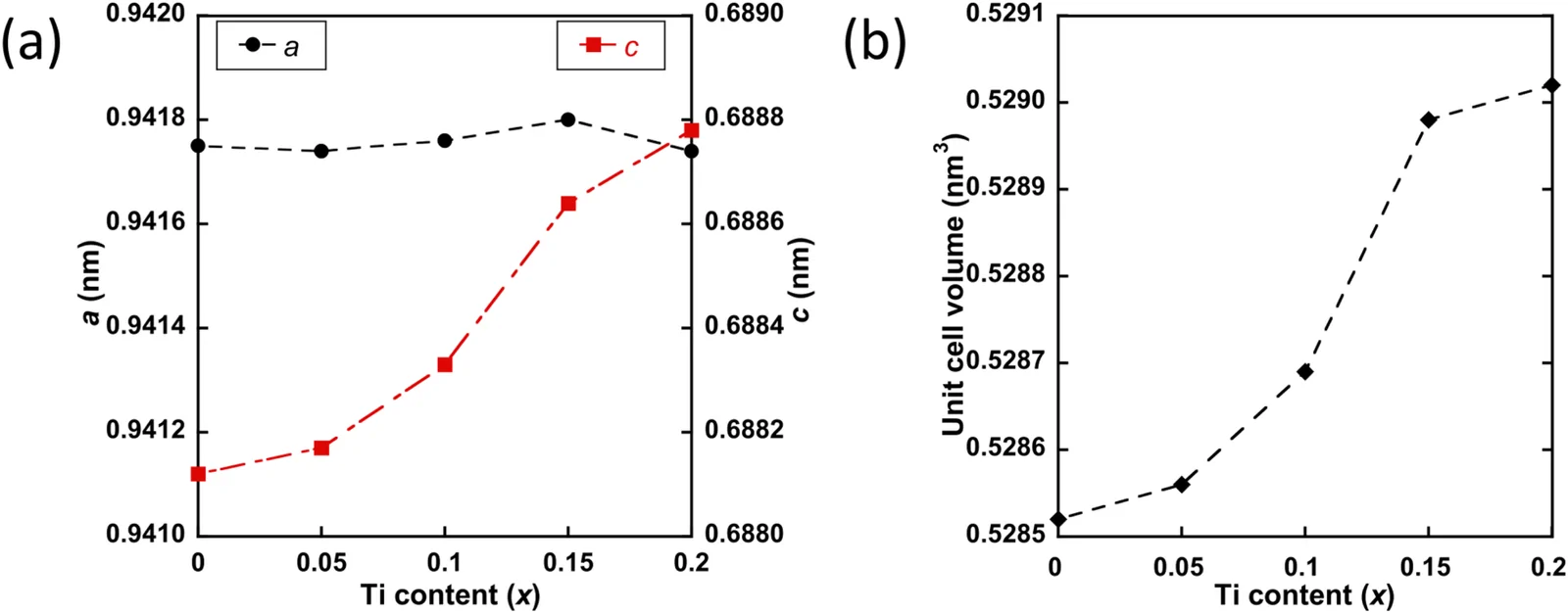
Lattice parameters (a) and unit cell volume (b) of Ti_*x*_-HAp obtained after heat treatment at 850 °C as functions of *x*.

Although the increase in lattice volume indicated incorporation of Ti into the channel, the behaviors in the lattice parameters, namely a nearly constant *a* and an increase in *c*, were observed when metal ions occupied either the 2b^40^ or 12i^36^ site located in the channel. To determine the site occupied by Ti, Rietveld refinement was performed on Ti_0.15_-HAp, which had the highest Ti content while retaining a single-phase structure. Fig. 3 shows the observed, calculated and difference curves in the final refinement in which a satisfactory convergence was yielded: *R*_wp_ and *R*_F_ were 3.906 and 0.964%, respectively. The refined structural parameters and the selected interatomic distances combined with bond angles are summarized in Tables 1 and S2, respectively. It should be noted here that the thermal displacement parameter *U* of Ti was fixed to the value shown in Table 1. The refined structure demonstrates that the site occupied by the incorporated Ti was the 2b site located at the center of the channel. It should be noted here that the refined structure did not converge to physically meaningful parameters when the structure was refined assuming that Ti occupied the 12i site. The refined site occupancy factor (SOF, *n*) of 0.088 was lower than the nominal value of 0.15. Given that the composition was almost identical among the multiple fields of view examined and no segregation of Ti-rich regions was observed (Table S3), the lower Ti content determined by Rietveld refinement than the nominal value was associated with segregation within the particles. Given that no diffraction lines or Raman bands attributable to Ti-containing species were detected in either the XRD pattern or the Raman spectra (see below), some Ti species may have been present in an amorphous form on the particle surface. The somewhat higher Ti content estimated by surface-sensitive SEM–EDS than the nominal value is thus likely associated with the presence of amorphous Ti species on the particle surface. The maximum content of Ti incorporated into the channel (0.088 per unit formula) was remarkably higher than that achieved in the previous report (2.5 × 10^−3^).^34^

**Fig. 3:**
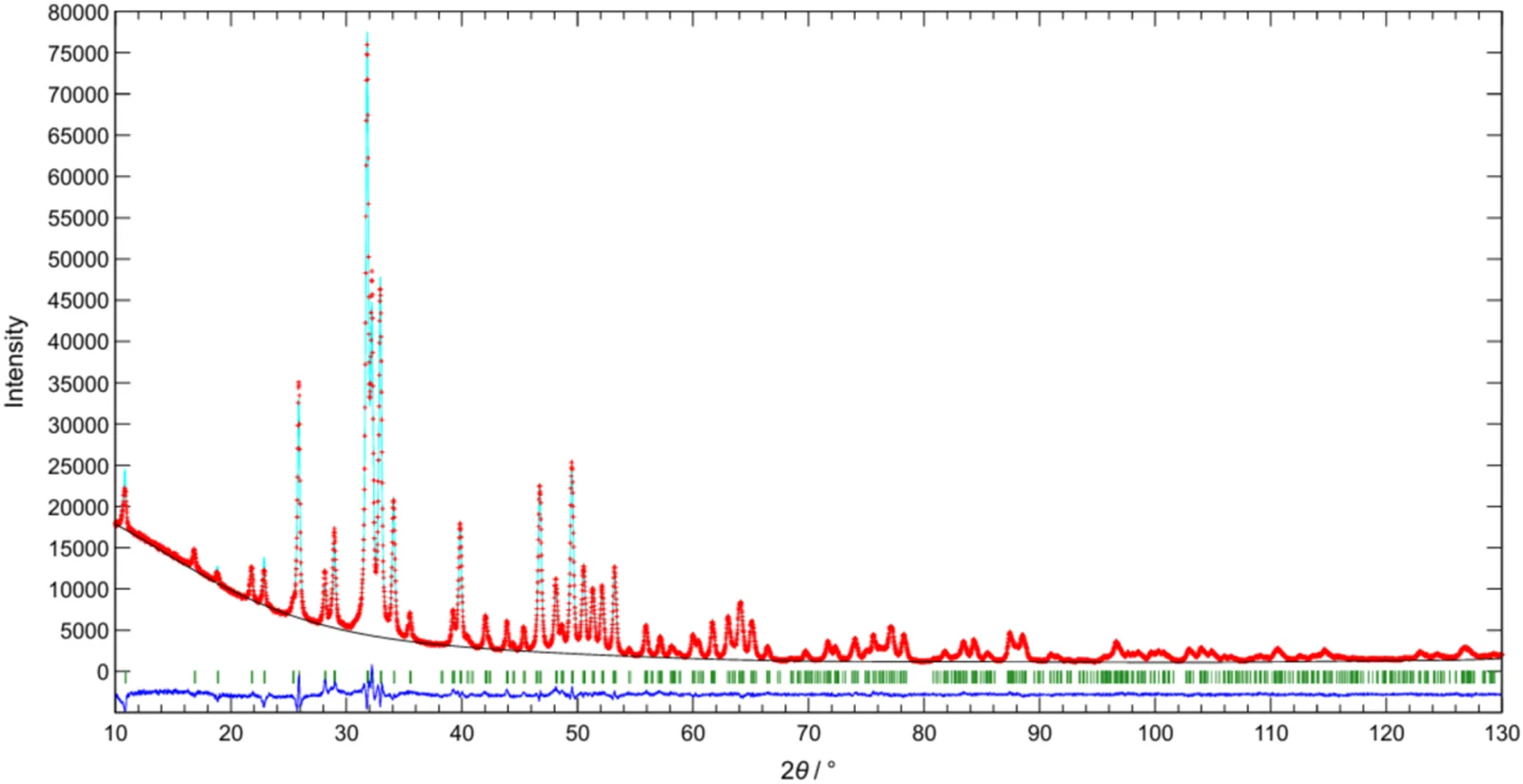
XRD pattern of Ti_0.15_-HAp; experimental (crosses) and calculated (solid line) curves of the Rietveld refinement. The bottom lines show the difference between the two curves. The positions of the apatite phase are shown as vertical lines.

**Table 1:** Refined structural parameters for Ti_0.15_-HAp

Space group; *P*6_3_/*m* (no. 176); *a* = 0.94198(22) nm, *c* = 0.68846(12) nm, *V* = 0.52904(19) nm^3^, *R*_wp_ = 3.906%, *R*_F_ = 0.964%, *S* = 2.7239							
Atom	Site	*g*	*n*	*x*	*y*	*z*	*U* (100 nm^2^)
O1	6h	1	6	0.3277(3)	0.4840(4)	1/4	0.0185(10)
O2	6h	1	6	0.5841(4)	0.4623(4)	1/4	0.0130(10)
O3	12i	1	12	0.3373(3)	0.2528(3)	0.0673(3)	0.0282(8)
O4	4e	0.5	2	0	0	0.1915(9)	0.0097(23)
P	6h	1	6	0.3973(2)	0.3686(2)	1/4	0.0112(4)
Ca1	4f	1	4	1/3	2/3	0.0018(3)	0.0107(4)
Ca2	6h	1	6	0.2455(1)	−0.0079(2)	1/4	0.0097(3)
Ti	2b	0.044(3)	0.088(6)	0	0	0	0.0152^a^

a Fixed during the final refinement.

Fig. 4 shows the IR spectra of Ti_*x*_-HAp and Ca_9.85_Ti_0.15_-HAp. In both cases, a band at 632 cm^−1^ attributable to the librational mode of OH^−^ in the channel of HAp was observed, in addition to absorption bands at 570, 600, and 960 cm^−1^ ascribable to PO_4_^3−^ vibrations.^49,50^ The intensity ratio of the hydroxyl- and phosphate-related absorption bands decreased with increasing *x* (Fig. S5), indicating that the number of OH^−^ decrease with increasing *x*. This result correlates well with the conversion of OH^−^ to O^2−^ upon metal cation incorporation into the channel for charge compensation.^41^ In addition, a new absorption band at 770 cm^−1^, which was not observed in the spectra of the parent HAp or Ca_9.85_Ti_0.15_-HAp, was observed in the case of Ti_*x*_-HAp (*x* > 0), and its intensity increased with increases in *x*. Given that similar bands in the 650–800 cm^−1^ range have been observed in the IR spectra of HAp containing Cu,^33,51,52^ Fe^36^ or Zn^40^ ions in the channel, the new band characteristics with respect to Ti_*x*_-HAp (*x* > 0) are assignable to Ti ions located in the channel.

**Fig. 4:**
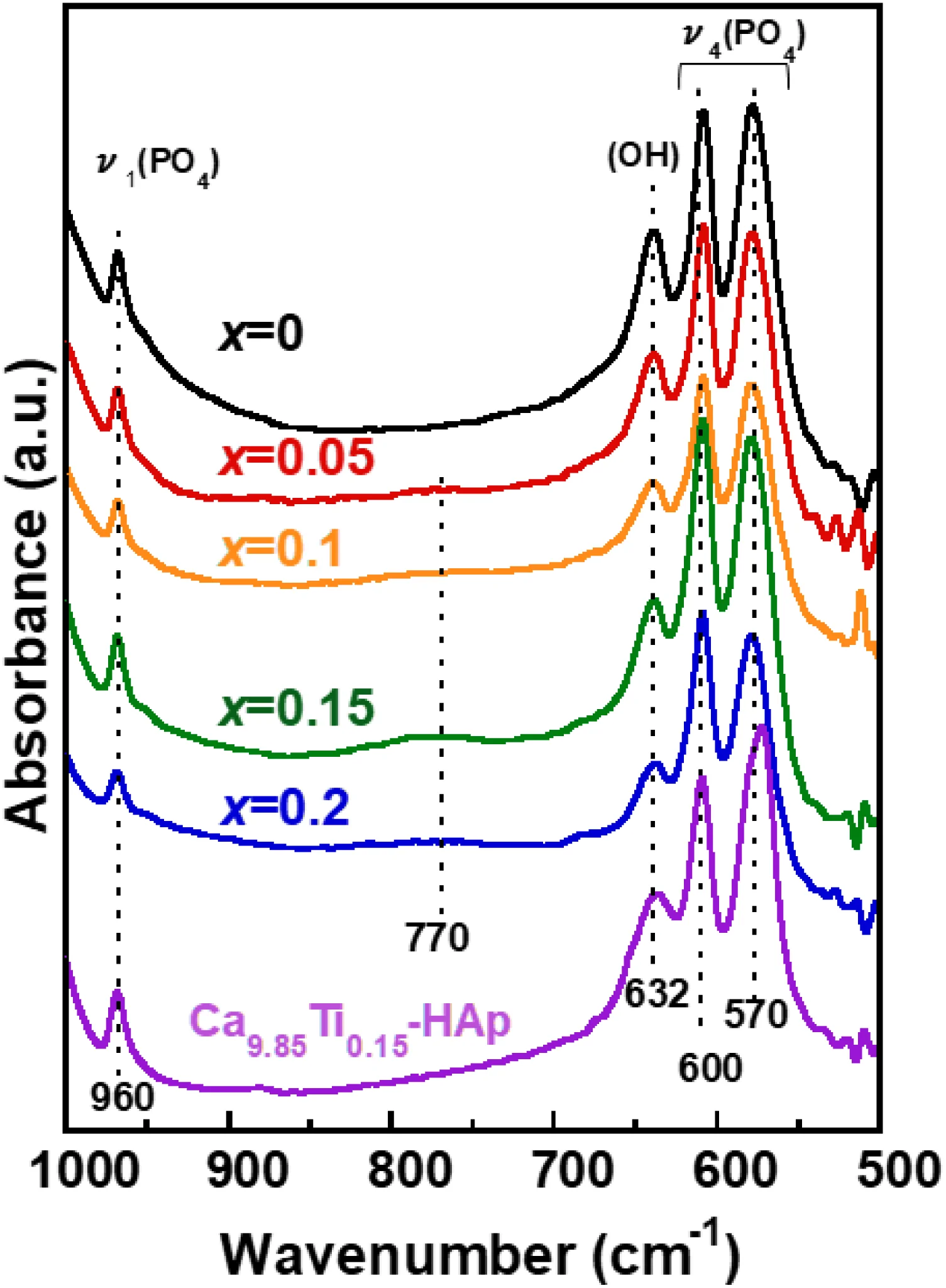
IR spectra of Ti_*x*_-HAp and Ca_9.85_Ti_0.15_-HAp.

The Raman spectra of Ti_*x*_-HAp and Ca_9.85_Ti_0.15_-HAp are shown in Fig. 5. In addition to the Raman bands ascribable to phosphate groups,^53,54^ that at 810 cm^−1^, which has not been reported for Ti-substituted HAp^55,56^ was observed in cases of Ti_*x*_-HAp, and its intensity increased with increases in *x*. It has been reported that Raman bands were observed at 630, 652 and 690 cm^−1^ in HAp containing Zn,^40^ Cu^37^ and Ni,^37^ respectively, and that these bands were attributable to [O–M–O] units based on the expected dependence of the vibrational frequency on atomic mass. Given that the mass of Ti is smaller than that of Ni, the fact that the absorption band observed in Ti_*x*_-HAp was located at a larger wavenumber (810 cm^−1^) when compared with that of Ni-incorporated HAp (690 cm^−1^)^37^ strongly indicates that the observed Raman band is ascribable to [O–Ti–O] units that formed between Ti at the 2b site and O^2−^ originating from OH^−^ in the channel. TiO_2_ polymorphs (anatase, rutile, or brookite)^57,58^ and CaTiO_3_ (ref. 59 and 60) were other possible candidates associated with the absorption band considering the composition, but the wavenumber value did not match that of any compound. The absorption bands at 450 and 610 cm^−1^, which are ascribable to CaTiO_3_,^61,62^ were observed in the spectrum for *x* = 0.2, further supporting the coexistence of CaTiO_3_ suggested by XRD analysis. As *x* increased, the peak at 960 cm^−1^ ascribable to the PO_4_^3−^ became broader, a result correlating with previous reports on local distortion of the phosphate groups upon metal incorporation into the channel.^29,36,38,40,46^

**Fig. 5:**
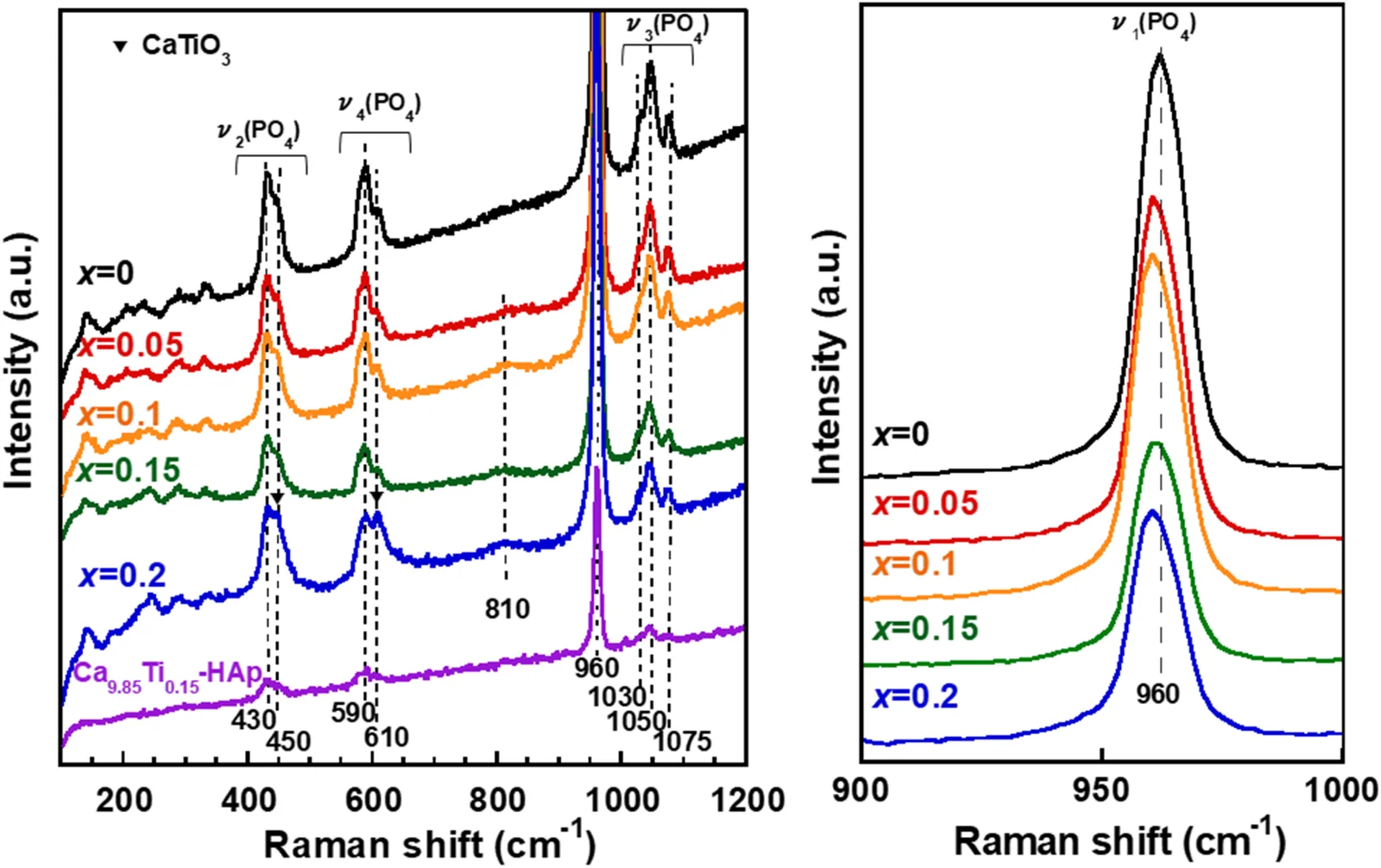
Raman spectra of Ti_*x*_-HAp and Ca_9.85_Ti_0.15_-HAp. An expanded version of the spectra is shown to the right.

Fig. 6a shows the UV-vis DR spectra of the products. While as-obtained HAp exhibited no characteristic absorption bands in any of the wavelengths, light absorption in the wavelength range of ≤350 nm was observed for Ca_9.85_Ti_0.15_-HAp, a trend correlating well with the previous report on development of UV-absorption characteristic in HAp upon Ti-substitution.^22,47^ In the case of HAp containing Ti in the channel, on the other hand, the intensity of the UV-absorption band was less prominent even when Ti with larger nominal content (*x* = 0.2) was loaded. Although the difference in particle size between Ca_9.85_Ti_0.15_-HAp and Ti_0.15_-HAp was suggested by the difference in crystallite size (17.5 and 31.1 nm for Ca_9.85_Ti_0.15_-HAp and Ti_0.15_-HAp, respectively), which was estimated from the main diffraction line using the Scherrer equation, the difference in spectral shape cannot be explained solely by particle size differences, but can rather be associated with the aforementioned alternatives in the local coordination environment. The fact that light absorption in the visible light range was negligible in both cases suggests that the introduced Ti existed in a tetravalent state, considering that introduction of Ti^3+^ into inorganic solids such as oxides and phosphates often resulted in development of visible light absorption.^63,64^ To confirm the oxidation state of Ti, the Ti 2p XPS spectrum of Ti_0.15_-HAp was measured (Fig. 6b). The Ti 2p_3/2_ and Ti 2p_1/2_ peaks were observed at binding energies of 458.4 and 463.7 eV, respectively. These values are in good agreement with those reported for Ti^4+^,^65,66^ further indicating that the incorporated Ti was present in the tetravalent state in the channel.

**Fig. 6:**
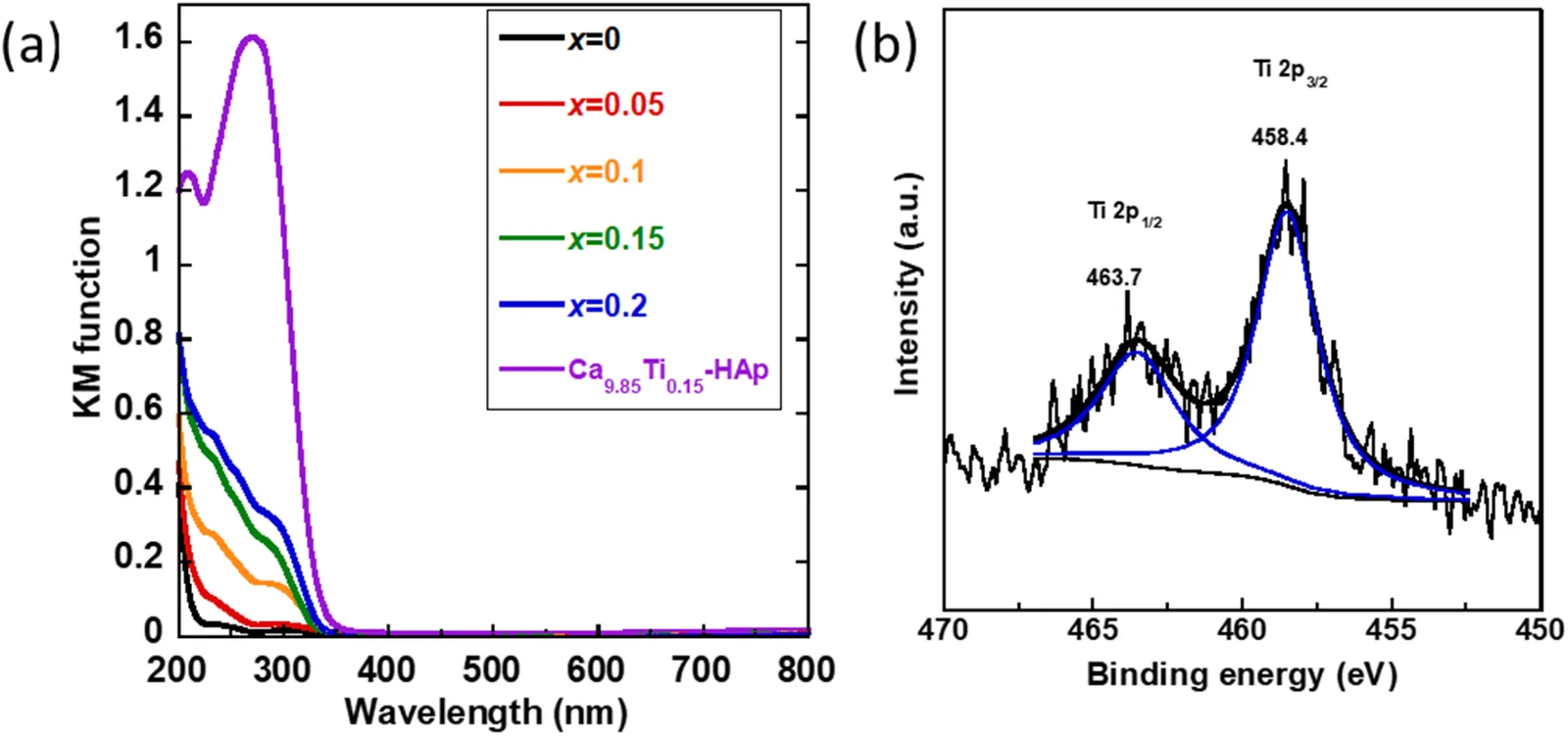
(a) UV-vis DR spectra of Ti_*x*_-HAp and Ca_9.85_Ti_0.15_-HAp, and (b) XPS spectrum of the Ti 2p region of Ti_0.15_-HAp.

To examine the deposition of Ti from the channel, Ti_0.15_-HAp was annealed in air. The XRD patterns of the Ti_0.15_-HAp after annealing are shown in Fig. 7a. While all the diffraction lines were assigned to the apatite phase in the cases of 800 and 900 °C, lines ascribable to Ca_3_(PO_4_)_2_ and CaTiO_3_ were additionally observed in the case of 1000 °C, indicating a collapse of the host apatite structure upon annealing at 1000 °C. Fig. 7b shows the Raman spectra of the products. In the case of 900 °C, in which no diffraction lines ascribable to compounds other than HAp were observed, an absorption band was observed at 680 cm^−1^ while all other bands were assigned to HAp.^53,54^ The intensity of the band became stronger when a higher annealing temperature (1000 °C) was employed, and the intensity of the band at 810 cm^−1^, which was attributable to [O–Ti^4+^–O] as discussed above, disappeared. These results suggest that the Raman band observed at 680 cm^−1^, which is characteristic of Ti_0.15_-HAp annealed at ≥900 °C, is associated with a change in the coordination environment of Ti. Considering that the band was not ascribable to any characteristic vibrational mode of TiO_2_ polymorphs (anatase, rutile, or brookite)^57,58^ or CaTiO_3_,^59,60^ however, the expected Ti deposition from the channel in the form of oxide was not confirmed.

**Fig. 7:**
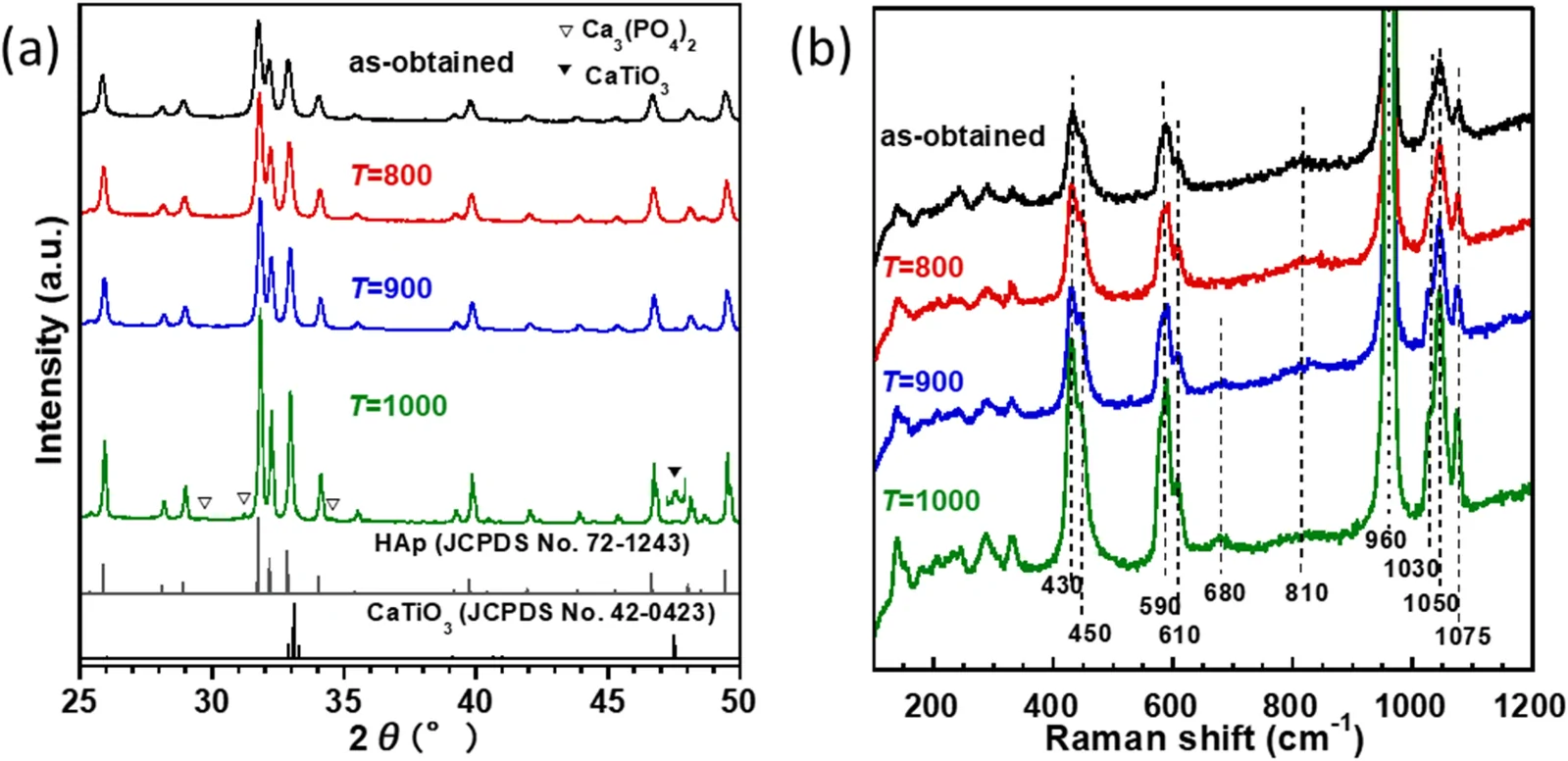
(a) XRD patterns and (b) Raman spectra of Ti_0.15_-HAp annealed at different temperatures.

## 4 Conclusions

Synthesis of hydroxyapatite containing tetravalent titanium (Ti^4+^) was verified with the aid of Rietveld refinement together with spectroscopic analyses performed on a product obtained by calcination of a hydroxyapatite impregnated with a titanium alkoxide. To the best of our knowledge, this study provides the first example of a hydroxyapatite in which the occupation of the channel sites by metal ions with a valence of four or higher has been confirmed by structural refinement. The hydroxyapatite containing Ti in the channel was distinguished from that substituted by Ti at the Ca site in terms of light absorption characteristics, a result correlating to the difference in the coordination environment of Ti. The insights gained in the present study suggest an expansion of hydroxyapatite from both the compositional and functional perspectives.

## Author contributions

Xiao Chen: investigation, writing – original draft, writing – review and editing. Kanji Saito: conceptualization, investigation, writing – original draft, writing – review and editing. Masataka Ogasawara: supervision, writing – review and editing. Sumio Kato: supervision, writing – review and editing.

## Conflicts of interest

There are no conflicts of interest to declare.

## Supplementary Material

RA-OLF-D6RA08560A-s001

## Data Availability

The data supporting this article have been included as part of the supplementary information (SI). Supplementary information is available. See DOI: https://doi.org/10.1039/d6ra08560a.
