# Supplementary material for: Incorporation of Ti4+ into the channel of hydroxyapatite by loading of Ti and post-calcination
Source: RSC Adv. 2026 Oct 26. Online ahead of print. doi: 10.1039/d6ra08560a (PMC13702838; doi:10.1039/d6ra08560a)
Supplement: Supplementary file 1 [file RA-OLF-D6RA08560A-s001.pdf]

Supplementary Information (SI) for RSC Advances.
This journal is © The Royal Society of Chemistry

Supporting Information

Incorporation of Ti^{4+} into the channel of hydroxyapatite by loading of Ti and post-calcination

Xiao Chen[a], Kanji Saito[a,b*], Masataka Ogasawara[a] and Sumio Kato[a*]

[a] Department of Materials Science, Graduate School of Engineering Science, Akita University, 1-1 Tegatagakuen-machi, Akita-shi, Akita 010-8502, Japan

[b] Kagami Memorial Research Institute for Materials Science and Technology, Waseda University, 2-8-26 Nishiwaseda, Shinjuku-ku, Tokyo 169-0054, Japan

Table S1 Lattice parameters and unit cell volumes of Ti_x-HAp and $Ca_{10-y}Ti_y$-HAp

sample	*a* (nm)	*c* (nm)	*V*(nm^3)
HAp	0.94175(3)	0.68812(2)	0.52852(3)
$Ti_{0.05}$-HAp	0.94174(11)	0.68817(8)	0.52856(10)
$Ti_{0.1}$-HAp	0.94176(10)	0.68832(7)	0.52869(9)
$Ti_{0.15}$-HAp	0.94180(5)	0.68864(4)	0.52898(5)
$Ti_{0.2}$-HAp	0.94174(16)	0.68878(12)	0.52902(16)
Ca_{10}-HAp	0.94305(9)	0.68872(7)	0.53045(9)
$Ca_{9.9}Ti_{0.1}$-HAp	0.94258(8)	0.68810(6)	0.52944(8)
$Ca_{9.85}Ti_{0.15}$-HAp	0.94254(11)	0.68792(8)	0.52926(11)

Table S2 Selected interatomic distances (nm) and bond angles (°) of $Ti_{0.15}$-HAp

Ca1-O1	0.2407(3)×3	Ca2-O1	0.2692(3)
Ca1-O2	0.2455(4)×3	Ca2-O2	0.2389(4)
Ca1-O3	0.2841(3)×3	Ca2-O3	0.2312(3)×2
P-O1	0.1525(5)	Ca2-O3	0.2497(3)×2
P-O2	0.1524(4)	Ca2-O4	0.2385(2)×2
P-O3	0.1574(3)×2	Ti-O4	0.1318(7)×2
Ti-O3	0.2901(3)	Ti-O4	0.2124(7)×2
O1-P-O2	111.8(2)	O1-P-O3	111.2(2)
O2-P-O3	108.2(2)	O3-P-O3	106.2(2)

Table S3 EDS-derived Ti/Ca molar ratios of $Ti_{0.15}$-HAp and $Ca_{9.85}Ti_{0.15}$-HAp

Sample	Entry	Element	wt %	mol %	Ti/Ca molar ratio
$Ti_{0.15}$-HAp	1	Ca	66.4	60.8	0.0194
		P	32.1	38.0	
		Ti	1.54	1.18	
	2	Ca	68.2	62.8	0.0191
		P	30.2	36.0	
		Ti	1.56	1.20	
	3	Ca	66.3	60.8	0.0248
		P	31.7	37.7	
		Ti	1.97	1.51	
$Ca_{9.85}Ti_{0.15}$-HAp	1	Ca	85.8	85.1	0.0167
		P	10.7	13.5	
		Ti	1.74	1.42	
	2	Ca	67.2	61.6	0.0180
		P	31.4	37.3	
		Ti	1.44	1.11	
	3	Ca	65.7	60.0	0.0198
		P	32.8	38.8	
		Ti	1.55	1.19	

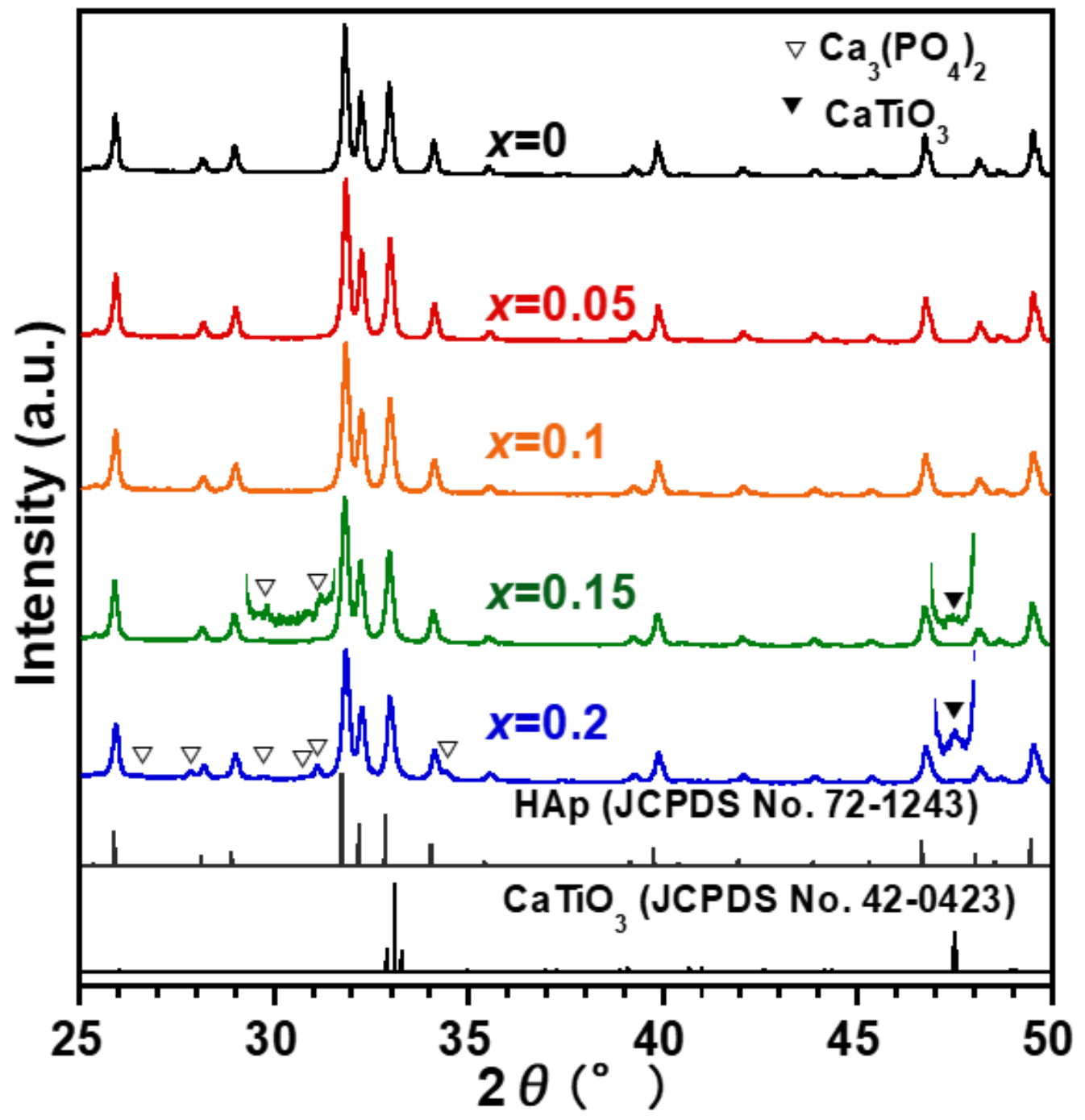

Fig. S1 XRD patterns of Ti_x-HAp obtained after heat treatment at 900 °C.

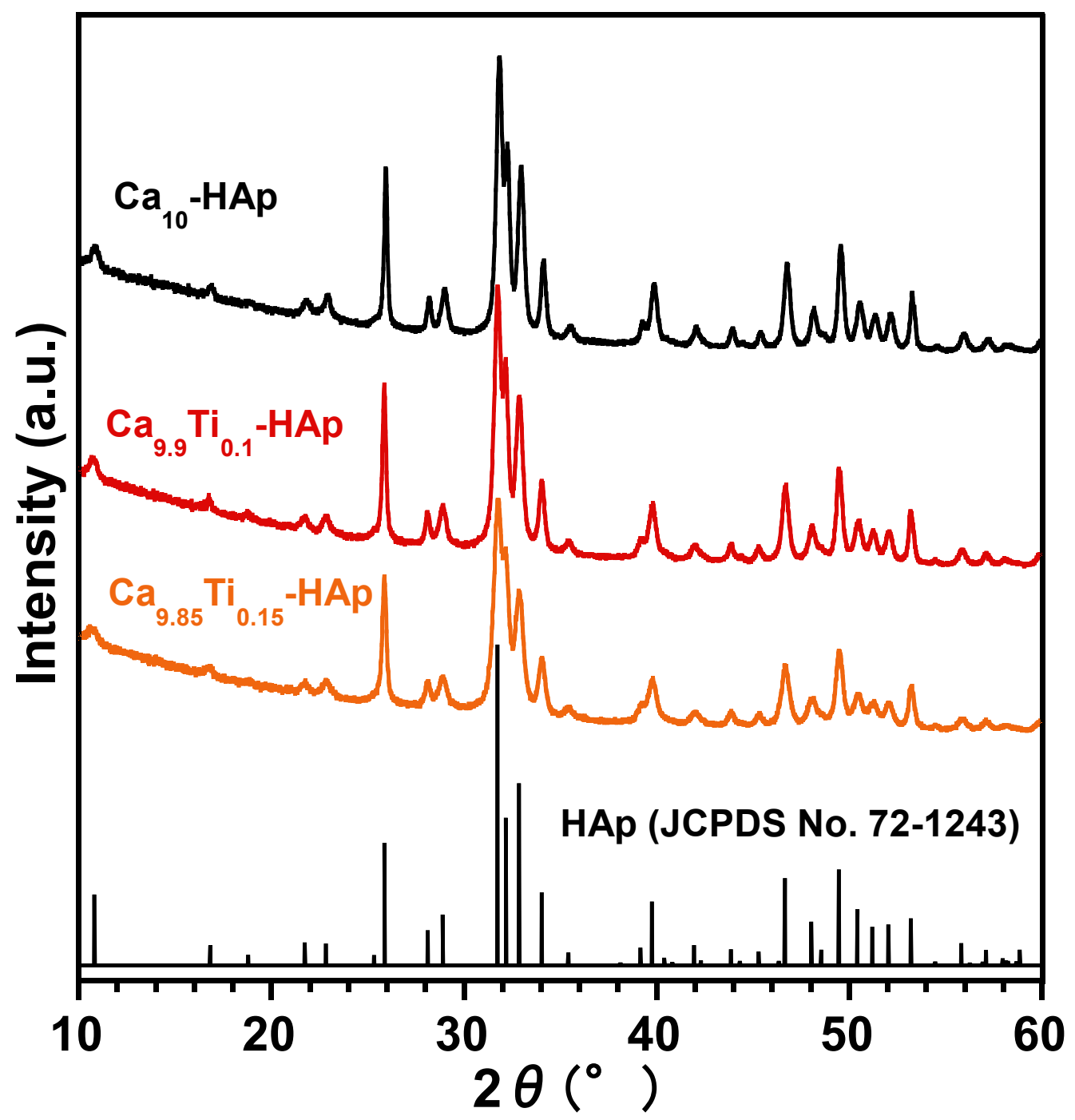

Fig. S2 XRD patterns of $Ca_{10-y}Ti_y$-HAp.

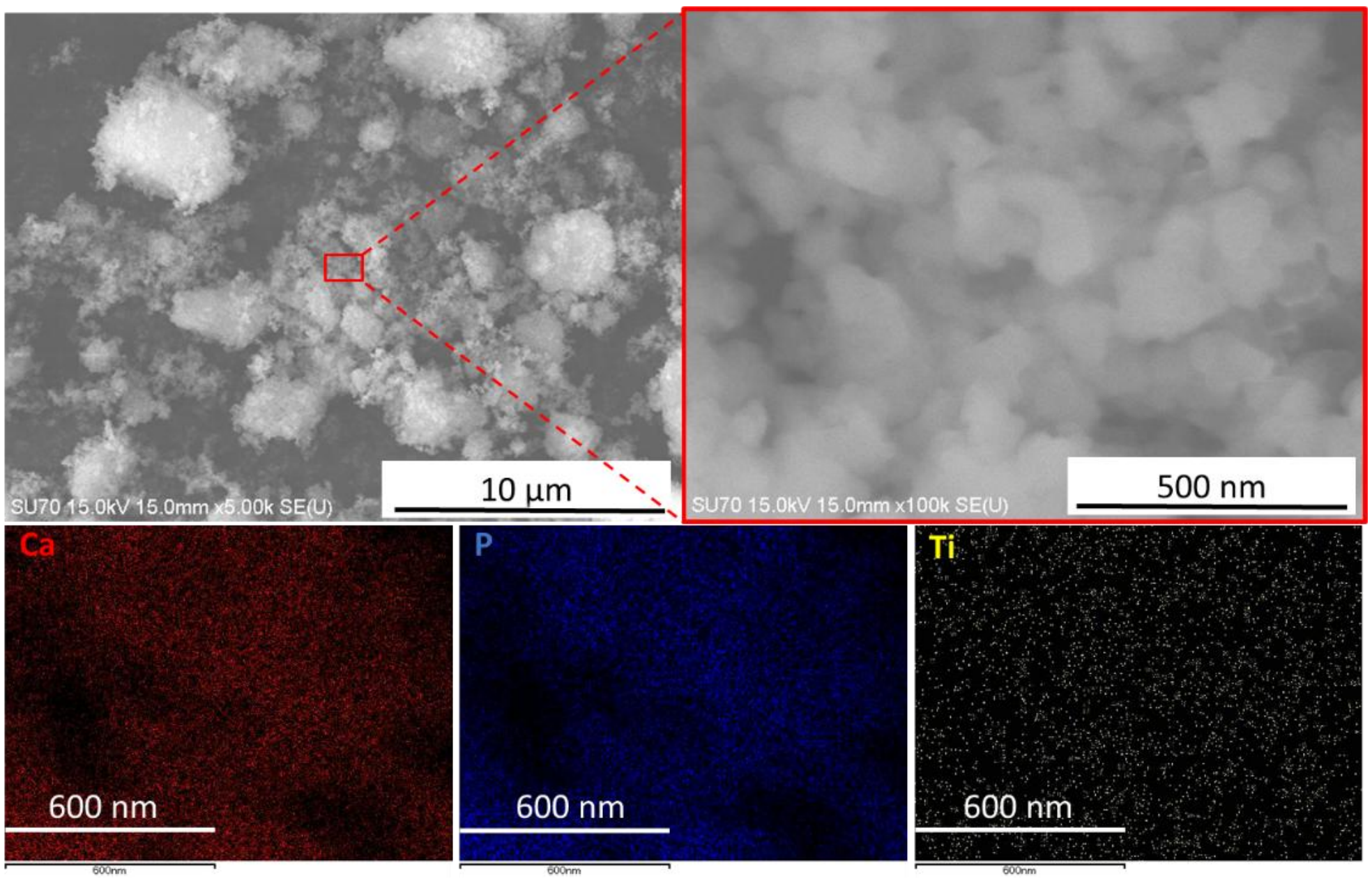

Fig. S3 FE-SEM images (top) and corresponding elemental mapping images (bottom) of $Ti_{0.15}$-HAp.

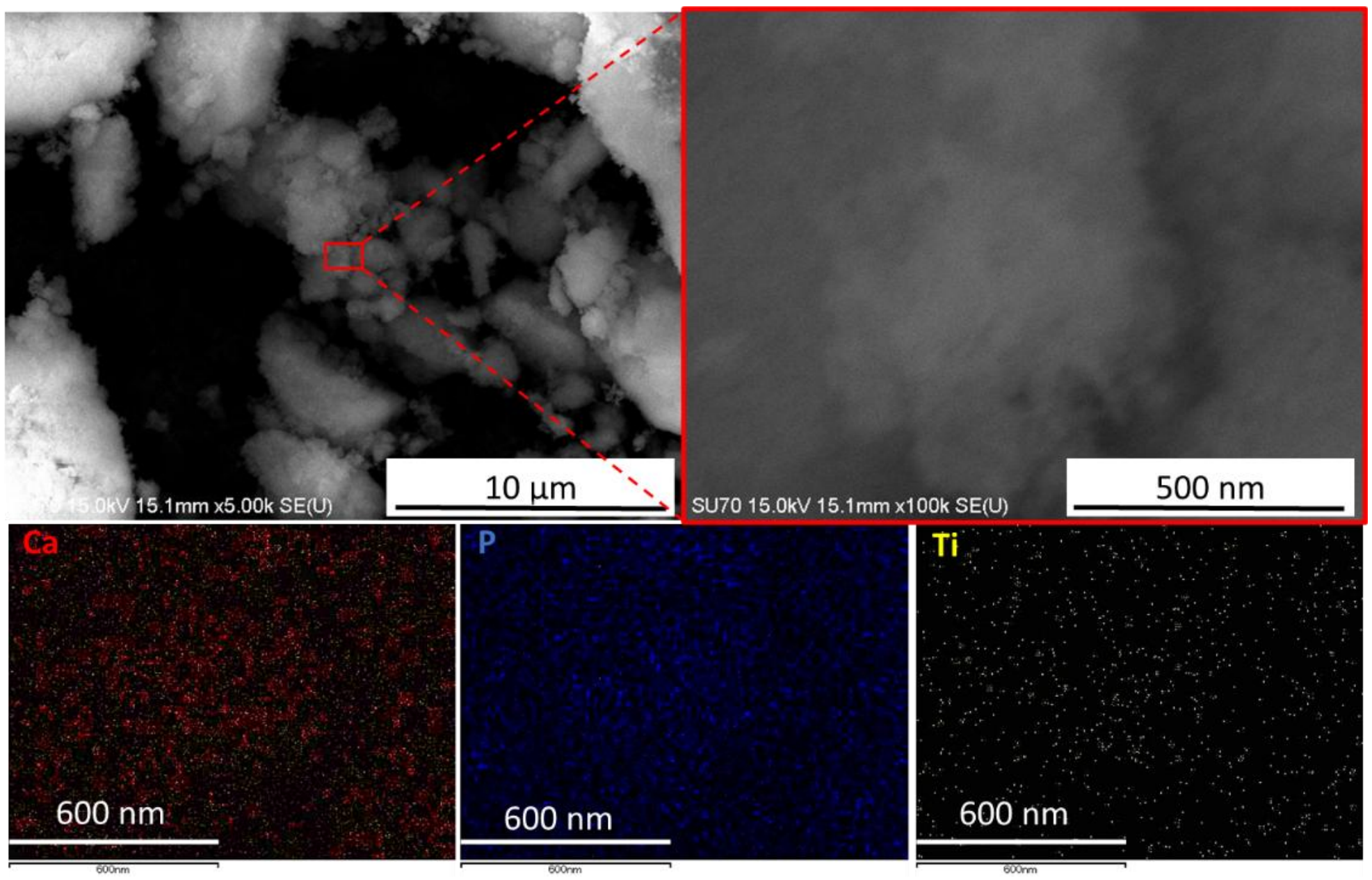

Fig. S4 FE-SEM images (top) and corresponding elemental mapping images (bottom) of $Ca_{9.85}Ti_{0.15}$-HAp.

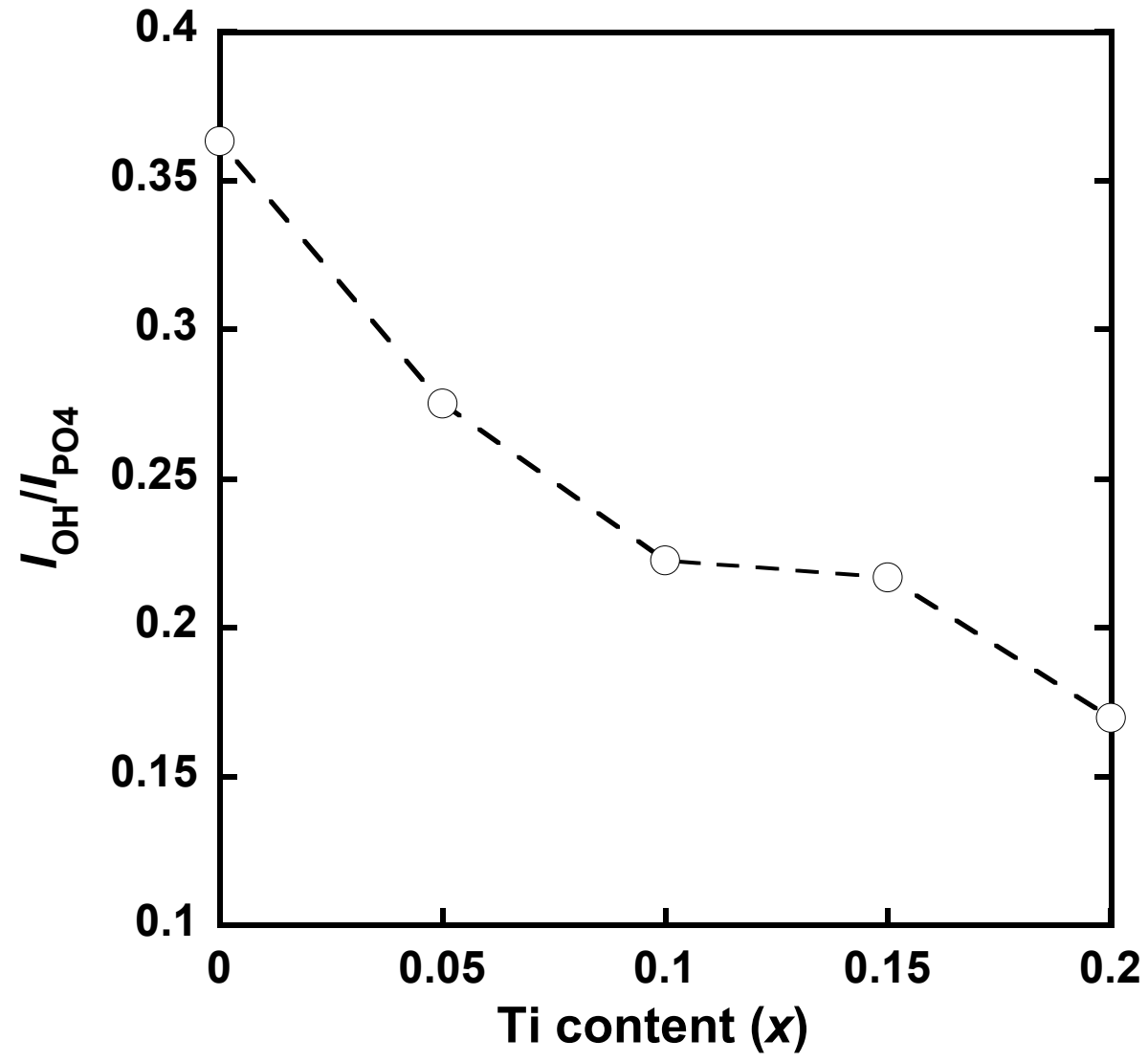

Fig. S5 Peak intensity ratio of the hydroxyl- to phosphate-related bands (I_{OH}/I_{PO4}) as a function of x. Peak intensities of OH^- (ca. 632 cm^{-1}) and PO_4^{3-} (ca. 600 cm^{-1}) were evaluated using baselines of 627–650 and 596–627 cm^{-1}, respectively.
